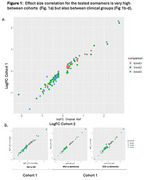# Microglial proteomic signature in human CSF allows stratification by APOE genotypes and is replicated in iPSC‐based model

**DOI:** 10.1002/alz70861_108563

**Published:** 2025-12-23

**Authors:** Alfredo Cabrera Socorro, Agustin Ruiz, Carlos Cruchaga, María Eugenia Sáez, Asif Emon, Bart Smets, Pablo García‐González, Raquel Puerta, Mercè Boada, Amanda Cano

**Affiliations:** ^1^ Johnson & Johnson Innovative Medicine, Beerse Belgium; ^2^ Joe R. and Teresa Lozano Long School of Medicine, University of Texas Health Science Center, San Antonio, TX USA; ^3^ Glenn Biggs Institute for Alzheimer’s & Neurodegenerative Diseases, University of Texas Health Science Center, San Antonio, TX USA; ^4^ Department of Neurology, Washington University School of Medicine, St. Louis, MO USA; ^5^ Washington University in St. Louis, School of Medicine, St. Louis, MO USA; ^6^ CAEBI. Centro Andaluz de Estudios Bioinformáticos, Sevilla Spain; ^7^ Johnson & Johnson Innovative Medicine, Germany, Wuppertal, Wuppertal Germany; ^8^ Ace Alzheimer Center Barcelona – International University of Catalunya (UIC), Barcelona Spain; ^9^ Ace Alzheimer Center Barcelona, Barcelona Spain

## Abstract

**Background:**

Amyloid‐beta (Aβ) and tau proteins have been historically used as hallmark of Alzheimer’s Disease pathology. Recently, High‐throughput proteomic platforms have been implemented to support better characterization of AD patients, for the discovery of novel biomarkers and to better understand disease biology and progression. Despite being the strongest genetic risk factor for Alzheimer’s Disease (AD), it is unknown if APOE genotype has impact the proteome in human cerebrospinal fluid (CSF).

**Method:**

We performed High Throughput proteomic profiling (SomaScan) of human CSF samples from two independent patient cohorts (1300 and 1147 samples, respectively). Differential expression (DE) analysis was performed to identify protein signatures associated with each APOE variant (APOE2 vs APOE3, APOE 4 vs APOE4 and APOE4 vs APOE3). Differentially expressed proteins were compared with the proteomic profile of LPS/myelin challenged iPSC‐derived microglial cells homozygous for all common APOE variants.

**Result:**

In a first cohort of 1300 CSF samples we identified a total of 83 proteins that were differentially expressed across APOE genotypes. 88.1% of these proteins were replicated in an independent cohort (*n* =1147 human CSF samples). Effect size of correlation was significant (Figure 1), and heterogeneity of samples and co‐variates did not impact replication (Figure 2). A significative number of CSF candidate proteins were replicated in microglial cell proteomics (table 3). Overall, there was concordance between the three experiments (untreated, myelin & LPS), although the largest number of replications were observed for myelin treated cells. Considering the 3 experimental settings, 18 proteins exhibited significant differential expression between genotypes in microglial cells with same direction of effect observed for CSF candidates (18 of 83, 21.7% of replicated proteins). It is noteworthy that four of these proteins have been previously identified as components of a 16‐protein signature associated with APOE genotypes in plasma samples and replicated in three independent studies (Sebastiani et al., 2019).

**Conclusion:**

Using proteomics as readout, this study demonstrates the translational value of iPSC‐derived models and proofs its value to assess mechanisms of function to better understand how APOE confers risk or protection for AD.